# Gene–Lifestyle Interactions in Renal Dysfunction: Polygenic Risk Modulation via Plant-Based Diets, Coffee Intake, and Bioactive Compound Interactions

**DOI:** 10.3390/nu17050916

**Published:** 2025-03-06

**Authors:** Meiling Liu, Da-Sol Kim, Sunmin Park

**Affiliations:** 1 Department of Chemical Engineering, Shanxi Institute of Science and Technology, Jincheng 048011, China; liumeiling@sxist.edu.cn; 2 Department of Food and Nutrition, Obesity/Diabetes Research Center, Hoseo University, 20 Hoseoro97bun-gil, BaeBang-Yup, Asan 41399, ChungNam-Do, Republic of Korea; tpfptm14@daum.net

**Keywords:** polygenic-risk score, glomerular filtration rates, plant-based diet, coffee, renal dysfunction

## Abstract

**Background:** This study aimed to investigate genetic variants associated with the estimated glomerular filtration rate (eGFR) and their interactions with lifestyle factors and bioactive compounds in large hospital-based cohorts, assessing their impact on renal dysfunction risk. **Methods:** Participants were categorized into two groups based on eGFR: High-GFR (control; *n* = 51,084) and Low-GFR (renal dysfunction; *n* = 7617), using an eGFR threshold of 60 mL/min/1.73 m^2^. Genetic variants were identified through a genome-wide association analysis, and their interactions with lifestyle factors were assessed a using generalized multifactor dimensionality reduction (GMDR) analysis. Additionally, interactions between polygenic risk scores (PRS) and nutrient intake were examined. **Results:** Low eGFR was associated with higher urinary protein levels (4.67-fold) and correlated with a Western-style diet and with saturated fat, arginine, and isoleucine intakes but not sodium intake. The genetic model for low eGFR included variants linked to energy production and amino acid metabolism, such as rs1047891_*CPS1*, rs3770636_*LRP2*, rs5020545_*SHROOM3*, rs3812036_*SLC34A1*, and rs4715517_*HCRTR2*. A high PRS was associated with a 1.78-fold increased risk of low eGFR after adjusting for sociodemographic and lifestyle factors. The PRS from the 6-SNP model interacted with plant-based diets (PBDs) and coffee intake, where individuals with higher PBD and coffee consumption had a lower risk of renal dysfunction. Additionally, *CPS1* rs1047891 interacted with vitamin D intake (*p* = 0.0436), where the risk allele was linked to lower eGFR with low vitamin D intake but not with high intake. Molecular docking showed that vitamin D3 had a lower binding energy to the CPS1 mutant type (−9.9 kcal/mol) than the wild type (−7.5 kcal/mol), supporting a potential gene–nutrient interaction influencing renal function. **Conclusions:** Middle-aged and elderly individuals with a high genetic risk for renal dysfunction may benefit from a plant-based diet, moderate coffee consumption, and sufficient vitamin D intake.

## 1. Introduction

Chronic kidney disease (CKD) affects approximately 10% of the global adult population, with the prevalence increasing steadily over the past two decades [1]. Among adults aged 35 years or older, CKD prevalence reaches approximately 14%, with higher rates observed in elderly populations due to demographic aging trends [1]. People of Asian descent appear to have a higher prevalence of CKD compared to other ethnicities, though this remains a subject of ongoing investigation [2]. CKD is strongly associated with metabolic syndrome (MetS), cardiovascular disease (CVD), cognitive dysfunction, and premature mortality. Importantly, CKD can be prevented through lifestyle modifications [3].

Renal function is measured as the filtration rate of fluid through the kidneys [4]. The gold standard measurement involves collecting a urinary volume over a 24 h period, which presents practical challenges in clinical settings [4]. Alternatively, creatinine—a breakdown product of creatine and creatine phosphate that freely passes through the glomerulus—is a useful biomarker. The creatinine clearance rate represents the glomerular filtration rate (GFR), a key indicator of kidney function. GFR correlates inversely with serum creatinine concentration and directly with urinary creatinine concentration and urine flow [4]. Significant nephron loss results in elevated serum creatinine levels [4]. Estimated GFR (eGFR) is calculated using serum creatinine levels, age, and sex to determine the blood filtration capacity per minute relative to the body surface area [5]. An eGFR ≤ 60 mL/min/1.73 m^2^ indicates reduced kidney function and potential CKD [6].

Multiple risk factors influence eGFR and CKD development, including aging, obesity, hypertension, impaired glucose tolerance, dyslipidemia, cognitive dysfunction, MetS, and CVD [7,8]. Various lifestyle factors also affect eGFR, though research on these factors remains limited. Smoking is a well-established lifestyle risk factor for reduced eGFR [7,8]. Other factors including sodium and potassium intake, alcohol and coffee consumption, and physical activity have been examined in cohort studies and meta-analyses, with inconsistent results [9]. High sodium intake (>4.6 g/day) has been associated with lower eGFR in some systematic reviews [7,8,9,10]. These inconsistencies may be attributable to genetic differences among populations.

Reduced kidney function reflects interactions between genetic and environmental factors [9,10]. Genes affecting eGFR may influence not only kidney function but also liver function, muscle mass, inflammation, and other physiological processes [11,12]. Ethnicity-related genetic differences are known to impact eGFR, with dietary factors—particularly high salt intake among Asians—interacting with genetic predispositions [13,14]. However, eGFR-related genetic variants remain understudied in certain Asian populations, and gene–lifestyle interactions require further investigation.

Exploring the direct metabolic effects of genetic variants in large cohort studies presents significant challenges. Consequently, molecular docking has emerged as a valuable approach for investigating gene–lifestyle interactions, especially for missense mutations affecting protein–ligand interactions. Previous research on obesity-related genetic variants such as brain-derived neurotrophic factor (*BDNF*) rs6265 has employed molecular docking to identify bioactive compounds influencing genetic risk [10]. Missense genetic variants influence renal function differently by interacting with bioactive compounds and lowering the binding energy. This study aims to identify the genetic variants associated with eGFR, analyze their interactions with lifestyle factors and bioactive compounds, and assess their impact on renal dysfunction risk in large hospital-based Asian cohorts.

## 2. Methods

### 2.1. Participants

This cohort study recruited individuals aged over 40 who visited multi-center hospitals in major Korean cities between 2010 and 2014. The study protocol received approval from the Institutional Review Board (IRB) of the Korea National Institute of Health and Hoseo University (approval numbers: KBP-2019-055 and 1041231-150811-HR-034-01). All participants provided written informed consent before enrollment.

### 2.2. Demographic, Anthropometric, and Biochemical Measurements

At baseline, participants completed comprehensive demographic questionnaires covering age, gender, education, income, and residential information. Trained research assistants performed anthropometric measurements, with the participants wearing light clothing and no footwear, following standardized protocols previously described.

Blood pressure was measured by a physician with participants in a seated position. Three consecutive readings were taken using a standard sphygmomanometer, and the mean values of systolic blood pressure (SBP) and diastolic blood pressure (DBP) were recorded for analysis [9].

Blood samples were collected after a minimum 12 h fasting period in tubes with and without heparin and ethylenediaminetetraacetic acid (EDTA) [9]. Fasting plasma glucose was analyzed using a Hitachi 7600 Automatic Analyzer (Hitachi, Tokyo, Japan), while hemoglobin A1c (HbA1c) concentrations were measured with an automatic analyzer (ZEUS 9.9; Takeda, Tokyo, Japan). Serum parameters, including total cholesterol, high-density lipoprotein cholesterol (HDL), triglycerides (TG), alanine aminotransferase (ALT), aspartate aminotransferase (AST), blood urea nitrogen (BUN), total bilirubin, and creatinine, were measured using an Hitachi 7600 Automatic Analyzer. High-sensitivity C-reactive protein (hs-CRP) concentrations were determined using a high-sensitivity ELISA kit (ThermoFisher, Waltham, MA, USA). Insulin resistance was calculated using the homeostatic model assessment of insulin resistance (HOMA-IR) [15,16].

Daily alcohol consumption (g/day) was calculated by assessing intake patterns of different beverage types (beer, wine, and spirits). For each beverage type, consumption frequency was multiplied by the average amount per intake, and these values were summed to determine total alcohol consumption [17]. Daily coffee intake was similarly calculated by multiplying consumption frequency by the amount per intake. Smoking status was categorized as follows: current smokers (≥20 cigarettes in the past six months), past smokers (>20 cigarettes in lifetime but none in the past six months), and never smokers [17]. Regular exercise was defined as engaging in moderate physical activity for more than 30 min on three or more days per week.

### 2.3. Assessment of Kidney Function

Kidney function was evaluated using the Modification of Diet in Renal Disease (MDRD) equation, as follows: 186 × serum creatinine concentration^−1.154^ × age^−0.203^ × 0.742 for women and 1 for men [18]. The MDRD equation was selected because the study population had a mean age of 53.7 years (SD = 8.0). This selection was supported by comparative studies of the MDRD and Chronic Kidney Disease Epidemiology Collaboration (CKD-EPI) equations across different age groups in a Chinese population, which found that the MDRD and CKD-EPI exhibited similar trends in eGFR, with the MDRD showing a reasonable decline in eGFR with age compared to the CKD-EPI [19]. The eGFR was standardized to a body surface area of 1.73 m^2^. Renal dysfunction was defined as an eGFR ≤ 60 mL/min/1.73 m^2^ or current use of kidney disease medication. Based on these criteria, participants were categorized into the following two groups: the Low-GFR group (*n* = 7617) and the High-GFR group (eGFR > 60 mL/min/1.73 m^2^, with no kidney disease medication; *n* = 51,084). The mean eGFR was significantly lower in the Low-GFR group (55.3 ± 0.34 mL/min/1.73 m^2^) than in the High-GFR group (87.1 ± 0.06 mL/min/1.73 m^2^), confirming an appropriate stratification of renal function between groups.

### 2.4. Usual Food Intake Assessment Using a Semi-Quantitative Food Frequency Questionnaire (SQFFQ)

The participants’ habitual food intake was evaluated using an SQFFQ specifically designed for the study population’s typical dietary patterns, covering the preceding 12 months [20]. The questionnaire comprised 106 commonly consumed food items, with participants documenting both consumption frequencies and portion sizes. Consumption frequencies were categorized as follows: never or seldom, once per month, two to three times per month, once or twice weekly, three or four times weekly, five or six times weekly, daily, twice daily, and three or more times daily. Portion sizes were recorded as 0.5, 1, or 2 times the standard portion size, which was visually represented through 106 food photographs. The SQFFQ’s reproducibility and validity were confirmed by comparison against three-day food records collected across all four seasons [21]. Daily food intake (g/day) was calculated by multiplying the median frequency of daily consumption by the reported portion size. Daily intake of energy, macronutrients (carbohydrates, proteins, amino acids, and fats), vitamins, and minerals were determined using Can-Pro 2.0 nutrient assessment software (Korean Nutrition Society, Seoul, Republic of Korea). Nutrient intake levels were categorized as low or high based on either established Dietary Reference Intake (DRI) cutoffs or study-specific percentiles, depending on the nutrient. For nutrients with well-defined DRI thresholds, these values were used to distinguish between low and high intakes. When no specific DRI cutoff was available or intake levels were highly variable, the 33rd and 66th percentiles of the study population’s intake distribution were used for classification according to the nutrients’ characteristics. For example, low vitamin D intake was defined as <2.53 µg/day, corresponding to the 33rd percentile in our study, rather than the DRI of 10 µg/day. The specific cutoff values for each nutrient are detailed in the corresponding tables and figure legends.

### 2.5. Dietary Pattern Classification and Inflammatory Index

Dietary patterns were classified using principal component analysis (PCA) based on 30 predefined food groups derived from the 106 individual food items. Using eigenvalues > 1.5 and orthogonal rotation (varimax), we identified the following four distinct dietary patterns: Asian-style balanced diet (ABD), plant-based diet (PBD), Western-style diet (WSD), and rice-based diet (RBD) [21,22]. Food groups with factor-loading values ≥ 0.40 were considered primary contributors to each pattern, as detailed in Supplemental Table S1.

The Dietary Inflammatory Index (DII) was calculated using a prediction equation incorporating food and nutrient intakes with corresponding dietary inflammatory weights [23]. This equation included energy, 32 nutrients, four food products, four spices, and caffeine [24]. Four components (garlic, ginger, saffron, and turmeric) were excluded from the DII calculation as they were not included in the SQFFQ. The final DII was computed as the sum of scores for the 38 included foods and nutrients, divided by 100.

### 2.6. Genotyping and Quality Control

Genomic DNA was extracted from participants’ whole blood samples and genotyped using a K-Chip (Affymetrix, Santa Clara, CA, USA) specifically designed to detect disease-related single-nucleotide polymorphisms (SNPs) in regional, rural, and urban hospital-based cohorts. Genotypes were imputed using 1000-genome sequence data or Asian HapMap data [25] and evaluated for accuracy using a Bayesian learning algorithm for robust general linear models [9,26]. Genotyping quality control was performed based on the following criteria: (1) genotyping accuracy ≥ 98%, (2) missing genotype call rate < 4%, (3) heterozygosity < 30%, and (4) absence of gender bias. Genetic variants were excluded from analysis if they failed to meet the threshold of *p* ≤ 0.05 for the Hardy–Weinberg equilibrium (HWE) or had a minor allele frequency (MAF) ≤1% [26].

### 2.7. Selection and Characterization of the Genetic Variants Associated with Renal Dysfunction Risk

The process of genetic variant selection and interaction analysis is illustrated in Figure S1. A genome-wide association study (GWAS) was conducted using the urban hospital-based cohort to identify genetic variants associated with renal function risk between the Low-GFR and High-GFR groups (significance threshold: *p* < 1 × 10^−6^). Genetic bias was assessed using a Manhattan plot and the genetic inflation factor (lambda) of the quantile-quantile (Q–Q) plot. The Manhattan plot displayed the negative logarithms of association *p*-values for renal function. The Q–Q plot evaluated GWAS genotype quality by comparing the quantile distribution of observed *p*-values (*y*-axis) against expected *p*-values (*x*-axis). Genotypes were considered ideal when the lambda value approached 1.

From an initial pool of 3787 genetic variants, 364 were excluded for failing to meet minor allele frequency (MAF < 1%) and Hardy–Weinberg equilibrium (*p* < 0.05) criteria, as determined using g:Profiler (https://biit.cs.ut.ee/gprofiler/snpense, accessed on 5 March 2024). Additionally, 3245 SNPs with high linkage disequilibrium (D’ ≥ 0.2) were excluded as they provided redundant genetic information. The remaining 178 genetic variants met the linkage disequilibrium criteria (D’ < 0.2), as assessed using Haploview 4.2 in PLINK. Another 103 variants were removed because of unidentifiable gene names, leaving 75 genetic variants. From these, we selected 22 genetic variants involved in a common genetic pathway identified using Genemania (https://genemania.org/, accessed on 29 March 2024). This pathway was linked to renal-function-associated genetic variants (*p* for Bonferroni correction <0.05) and was selected using MAGMA gene-set analysis in SNP2GENE of the Functional Mapping and Annotation of Genetic Associations (FUMA) web application, available through the git repository (https://github.com/Kyoko-wtnb/FUMA-webapp/, accessed on 10 April 2024).

An expression quantitative trait locus (eQTL) analysis was employed to directly assess how genetic variants at risk loci influence candidate gene expression. This approach helped determine the gene expressions associated with genetic variants linked to hypo-HDL risk using the Genotype-Tissue Expression (GTEx) × eQTL calculator (https://gtexportal.org/home/testyourown, accessed on 30 April 2024).

### 2.8. PRS Development and Gene–Lifestyle Interactions

From the 22 SNPs identified through the generalized multifactor dimensionality reduction (GMDR), we selected 10 for the best model of SNP–SNP interactions related to renal dysfunction risk. Selection criteria for the optimal model included *p* < 0.001 for the sign test of testing-balanced accuracy (TEBA) and 10-fold cross-validation consistency (CVC) in the exhaustive search type after covariate adjustment. The following two adjustment models were considered: model 1 (covariates: age, gender, residence area, education, and income) and model 2 (model 1 covariates plus energy, sodium, and alcohol intake; regular exercise; and smoking status). Ten-fold cross-validation was employed because of the large sample size (>1000) [27].

We calculated the polygenic risk score (PRS) by summing the number of risk alleles from each selected SNP in the best gene–gene interaction model. Risk allele values were assigned as follows: homozygous risk genotype (for example, “TT”) = 2; heterozygous genotype (for example, “TC”) = 1; and homozygous non-risk genotype (for example, “CC”) = 0. The PRS values were categorized into Low-PRS (0–4 for the 6-SNP model; 0–5 for the 7-SNP model), Medium-PRS (5–6 for the 6-SNP model; 6–7 for the 7-SNP model), and High-PRS (>6 for the 6-SNP model and >7 for the 7-SNP model). We selected the model with the fewest SNPs for the lifestyle interaction analyses.

### 2.9. Molecular Docking Analysis of Missense Mutation with Food Compounds

Both wild-type and mutated protein structures were obtained in the Protein Data Bank (PDB) format via the Iterative Threading Assembly Refinement (I-TASSER) website (https://zhanggroup.org/I-TASSER/, accessed on 14 May 2024). These protein structures were converted to PDB, partial charge (Q), and atom type (T) (PDBQT) files using AutoDock Tools 1.5.6, provided by the Molecular Graphics Laboratory at the Scripps Research Institute (La Jolla, CA, USA) [28]. Active sites within the proteins were identified using ProteinsPlus (https://proteins.plus/, accessed on 29 May 2024), with both active functional pockets and mutated sites included in the active site for molecular docking. Concurrently, approximately 20,000 food compounds were transformed into the PDBQT file format, with any water molecules attached to the ligands removed [28]. Food components were selected based on binding energy thresholds of less than −10 kcal/mol [29]. Lower binding free energy values indicate a lower binding energy between the protein and ligand, suggesting a more stable interaction.

### 2.10. Statistical Analysis

Statistical analyses were conducted using SAS (version 9.3; SAS Institute, Cary, NC, USA). The study included 58,701 participants, providing sufficient statistical power to detect significance at α = 0.05 and β = 0.99, as well as an odds ratio (OR) of 1.05 in the logistic regression analysis, as determined by the G-power calculation.

For the categorical variables, frequency distributions were calculated, and chi-square tests assessed statistical differences in low eGFR (eGFR ≤ 60 mL/min/1.73 m^2^). For continuous variables, adjusted means with standard errors were calculated after controlling for covariates, including age, gender, residence area, education, income, daily energy intake, sodium intake, alcohol consumption, smoking status, and physical activity. Differences between the Low-GFR and High-GFR groups were evaluated using one-way analysis of covariance (ANCOVA) with covariate adjustment [30]. When ANCOVA revealed significant differences, Tukey’s test was applied for post hoc multiple comparisons.

The association between low eGFR and metabolic parameters was examined through logistic regression with covariate adjustment. The results are presented as an adjusted OR with the 95% confidence interval (CI) for each metabolic parameter. The following two adjustment models were implemented: Model 1, including age, residence area, survey year, education, and income as covariates; Model 2, incorporating all Model 1 covariates plus energy intake, alcohol consumption, sodium intake, physical activity, and smoking status.

Interactions between PRS or carbamoyl-phosphate synthase-1 (*CPS1*) rs1047891 (Thr1406Asn in protein) and lifestyle-related parameters were analyzed using two-way ANCOVA. Participants were categorized into high or low groups based on dietary reference intake values or the 66th percentiles of each lifestyle variable. Within these high and low lifestyle parameter groups, the OR and 95% CI for the low eGFR were determined using logistic regression analysis. Additionally, chi-square tests assessed statistical differences in low eGFR prevalence across PRS groups within the stratified lifestyle parameter categories.

## 3. Results

### 3.1. Demographic and Lifestyle Characteristics

Table 1 presents the general characteristics of participants stratified by renal function. Participants in the Low-GFR group were significantly older than those in the High-GFR group, with older individuals having a 2.16 times higher risk of renal dysfunction. Interestingly, the female gender showed an inverse association with renal dysfunction compared to males. Educational attainment did not significantly differ between the groups, indicating no association between education level and eGFR (Table 1). While the Low-GFR group contained more former and current smokers than the High-GFR group, smoking status showed no significant association with eGFR after covariate adjustment (Table 1).

### 3.2. Biochemical Parameters Related to Metabolic Syndrome

MetS prevalence was significantly higher in the Low-GFR group compared to the High-GFR group (Table 1). Serum creatinine concentration demonstrated strong positive correlations with both blood urea nitrogen (r^2^ = 0.3595, *p* < 0.0001) and uric acid (r^2^ = 0.475, *p* < 0.0001). Individuals with MetS had a 1.991-fold higher risk of renal dysfunction (Table 1). The association between CVD and renal dysfunction followed a similar pattern to that observed with MetS. Anthropometric measures including body mass index (BMI) and waist circumference were significantly elevated in the Low-GFR group compared to the High-GFR group, with adjusted odds ratios positively associated with low eGFR (1.37 for BMI and 1.30 for waist circumference; Table 1). Metabolic parameters including fasting serum glucose, HbA1c, LDL cholesterol, and triglycerides were all significantly higher in the Low-GFR group and demonstrated positive associations with low eGFR, with odds ratios ranging from 1.277 to 1.853 (Table 1). Conversely, serum HDL cholesterol showed an inverse association with low eGFR (OR = 0.66; Table 1).

Although systolic blood pressure (SBP) and diastolic blood pressure (DBP) did not differ significantly between the Low-GFR and High-GFR groups, SBP showed a positive association with eGFR (Table 1). Hypertension prevalence was significantly higher in the Low-GFR group, with hypertensive individuals having a 2.09 times higher risk of developing renal dysfunction (Table 1). Insulin resistance, as determined by HOMA-IR, was also positively associated with low eGFR (OR = 1.47; Table 1). The inflammatory marker high-sensitivity C-reactive protein (hs-CRP) was significantly lower in the High-GFR group, and individuals with elevated hs-CRP (>0.5 mg/dL) had a 1.71-fold higher risk of renal dysfunction (Table 1).

Beyond the MetS parameters, eGFR was also associated with markers of liver function. Serum uric acid, BUN, and total bilirubin concentrations were substantially higher in the Low-GFR group compared to the High-GFR group, with strong positive associations with low eGFR (OR = 2.43–4.53; Table 1). Serum albumin concentrations were lower in the Low-GFR group and inversely associated with low eGFR (Table 1). Among liver enzymes, only serum alanine aminotransferase (ALT) activity showed a positive association with eGFR (Table 1).

### 3.3. Lifestyles and Nutrient Intake

Participants in the Low-GFR group demonstrated significantly lower energy intake than those in the High-GFR group. Carbohydrate and protein intakes did not differ significantly between the Low-GFR and High-GFR groups, and neither was associated with low eGFR (Table S1). Despite similar total protein intake between groups, both essential amino acid and animal protein consumption were significantly higher in the Low-GFR group, showing positive associations with low-GFR. However, the amino acid analysis revealed that participants in the Low-GFR group consumed higher amounts of arginine and isoleucine than the High-GFR group, with these amino acids positively linked to low-GFR. Conversely, cysteine intake demonstrated an inverse association with low eGFR (Table S1). Fat intake was significantly higher among participants in the Low-GFR group than the High-GFR group and showed an inverse relationship with low eGFR. However, no significant differences were observed between groups regarding saturated, monounsaturated, and polyunsaturated fatty acid consumption. Sodium intake was similar between the Low-GFR and High-GFR groups and showed no association with low eGFR (Table S1). Several micronutrients and dietary quality indicators including calcium, vitamin D, vitamin C, flavonoid intake, and DII showed no significant differences between groups and were not associated with low eGFR (Table S1). Among the four dietary patterns analyzed, WSD consumption was significantly higher in the Low-GFR group compared to the High-GFR group, with WSD intake positively associated with low eGFR. The other dietary patterns—ABD, PBD, and RMD—showed no association with low eGFR (Table S1).

Alcohol consumption was significantly lower in the Low-GFR group compared to the High-GFR group and demonstrated an inverse association with renal dysfunction (Table S1). Similarly, coffee intake was lower among participants in the Low-GFR group and showed an inverse association with low eGFR. In contrast, tea consumption did not differ between groups and showed no association with eGFR. While the proportion of individuals engaging in regular exercise was higher in the Low-GFR group than in the High-GFR group, no significant association was observed between regular exercise and eGFR after covariate adjustment (Table S1).

### 3.4. Polygenic Variants and Their Interaction Related to Renal Dysfunction Risk

The GWAS revealed multiple genetic variants associated with renal dysfunction as measured by eGFR. The Manhattan plot (Figure S2A) illustrates the statistical associations of the genetic variants across all chromosomes. The Q–Q plot demonstrated good consistency between observed and expected *p*-values, with a genome inflation factor (λ) of 1.054, confirming no significant inflation in the GWAS results (Figure S2B).

Following our selection criteria (*p* < 1 × 10^−6^ for GWAS, D’ < 0.2 for linkage disequilibrium, *p* ≥ 0.05 for Hardy–Weinberg equilibrium, and minor allele frequency ≥0.01), we identified 22 SNPs associated with renal dysfunction. Among these, the following 10 genetic variants were selected based on genetic variant–genetic variant interaction analysis: rs1047891_*CPS1*, rs3770636_LDL receptor-related protein-2 (*LRP2*), rs5020545_Shroom family member-3 (*SHROOM3*), rs3812036_solute carrier family 34 member-1 (*SLC34A1*), rs4715517_hypocretin receptor-2 (*HCRTR2*), rs140652052_BRI1-associated kinase 1 (*BAK1*), rs139132767_suppression of tumorigenicity 7 (*ST7*), rs141574969_coiled-coil domain containing 63 (*CCDC63*), rs1031755_WD repeat domain 72 (*WDR72*), and rs6001939_myocardin-related transcription factor A (*MRTFA*) (Table 2). All variants demonstrated significant associations with renal dysfunction risk in the city hospital-based cohort (*p* < 5 × 10^−7^). Three variants—rs3770636 (*LRP2*), rs1031755 (*WDR72*), and rs6001939 (*MRTFA*)—showed inverse associations with low eGFR, while the remaining seven variants were positively associated with increased risk (Table 2). Most identified variants were located in intronic regions. Notably, rs4715517 (*HCRTR2*) was found in the 5’-untranslated region (UTR) and rs140652052 (*BAK1*) in the nonsense-mediated decay (NMD) transcripts, with rs1047891 (*CPS1*) representing a missense variant (Table 2).

The eQTL analysis revealed that the risk allele (T) of *SHROOM3*_rs5020545 was associated with significantly reduced gene expression compared to the non-risk allele (C) in liver tissue (coefficient: −0.2, *p* = 0.00015). Conversely, the risk allele (A) of *CPS1*_rs1047891 exhibited a markedly increased expression relative to the non-risk allele (C) specifically in the hypothalamus.

GMDR analysis identified models with 6–10 SNPs that met the sign test criteria for TEBA (*p* < 0.05) and CVC (10/10), indicating significant interactions among genetic variants influencing low eGFR (Table S2). The six-SNP model included *LRP2*_rs3770636, *CPS1*_rs1047891, *SHROOM3*_rs5020545, *CCDC63*_rs141574969, *SLC34A1*_rs3812036, and *MRTFA*_rs6001939. The seven-SNP model incorporated these six variants plus *WDR72*_rs491567 (Table S2).

**Figure 1:**
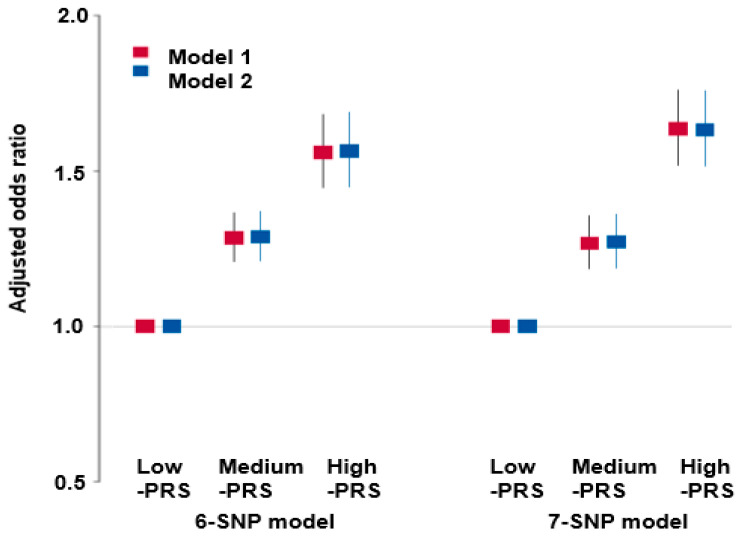
Adjusted odds ratio (OR) and 95% confidence intervals (CI) of 6-SNP PRS and 7-SNP PRS for renal dysfunction risk measured by estimated glomerular filtration rates. PRS was generated with the sum of the number of risk alleles in each genetic variant and classified into Low-PRS, Middle-PRS, and High-PRS, respectively, in the 6- and 7-SNP models. Low-PRS (0–4; 0–5), Medium-PRS (5–6; 6–7), and High-PRS (>6; >7) in 6-SNP and 7-SNP models, respectively. SNP: Single-nucleotide polymorphism; PRS: Polygenic risk score.

PRS was calculated to assess the predictive value of selected models. The six-SNP model showed significant association with low eGFR, with odds ratios of 1.761 (95% CI: 1.526–1.984) and 1.772 (95% CI: 1.509–2.020) in models 1 and 2, respectively, after adjusting for different covariates (Figure 1). Similarly, the seven-SNP model yielded odds ratios of 1.737 (95% CI: 1.480–1.996) and 1.782 (95% CI: 1.492–2.061) in models 1 and 2, respectively (Figure 1). These results suggest that the six-SNP model represents the optimal model for predicting renal dysfunction risk.

### 3.5. Metabolic Functions Related to Genes Involved in the Hypo-eGFR

Functional analysis revealed that genes associated with low eGFR were linked to multiple biological processes and pathways, including energy production through ATP synthase activity; protein autoprocessing; regulation of receptor recycling; liver functions, such as liver cancer, thiazolidinedione regulation, LDL pathways, estrogen receptor 1 (*ESR1*) targets, and insulin signaling; and skeletal muscle function through myosin activity, neuromuscular process, and oligodendrocyte differentiation (Table 3). Notably, the identified genes were not directly associated with renal filtering processes but rather with ATP-producing pathways and the production of nitrogen-composed waste from the liver and skeletal muscles (Table 3).

### 3.6. Genetic Variants-Lifestyle Interaction with Hypo-eGFR

**Figure 2:**
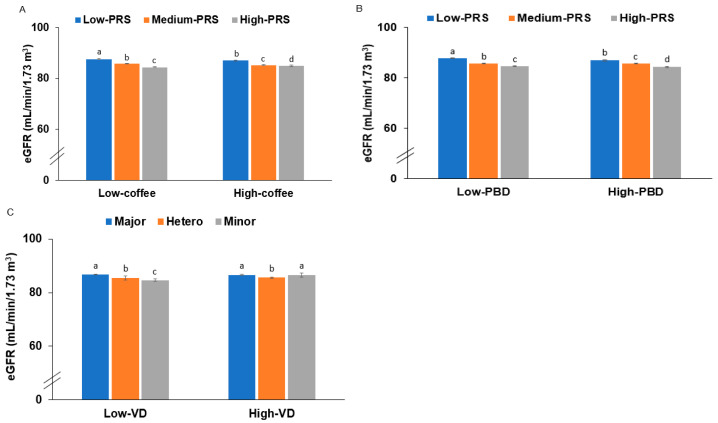
Estimated glomerular filtration rates (eGFRs) according to the polygenic risk scores (PRS) of the 6-SNP model or CPS1 alleles and different lifestyles: (**A**) plant-based diet and PRS (cutoff: 66th percentile); (**B**) coffee intake and PRS (cutoff: 3 g/day); (**C**) vitamin D3 intake and CPS1 WT and MT (cutoff: 2.53 μg/day; 33rd percentile). Low-PRS (0–4), Medium-PRS (5–6), and High-PRS (>6) in the 6-SNP model. ^a,b,c^ Different letters on the bars indicate significant differences among the PRS groups in Tukey’s test at *p* < 0.05.

An analysis of the interactions between the PRS of the 6-SNP model and lifestyle factors revealed significant interactions with PBD (*p* = 0.0415) and coffee intake (*p* = 0.0092) in relation to low eGFR risk (Table 4). Participants with higher consumption of PBD and coffee demonstrated an attenuated association between PRS and low eGFR, suggesting potential protective effects of these dietary factors (Figure 2A,B). In contrast, no significant interactions were observed between PRS and either alcohol consumption or regular exercise concerning renal dysfunction risk (Table 4).

Further analysis identified a significant interaction between *CPS1* rs1047891 and vitamin D intake (*p* = 0.0477; Table 4). Specifically, individuals carrying the risk allele of *CPS1* rs1047891 exhibited lower eGFR when vitamin D intake was low, whereas this adverse effect was substantially mitigated among those with high vitamin D intake (Figure 2C).

### 3.7. Bioactive Compound Interaction with CPS1 rs1047891 Missense Mutation (Thr1406) and Molecular Docking

To investigate the potential molecular mechanisms underlying the observed gene–diet interactions, we conducted molecular docking analyses of approximately 1100 polyphenol compounds from vegetables and fruits, including anthocyanidins, flavonoids, carotenoids, and saponins, with the CPS1 protein. Our analysis identified 19 bioactive compounds that exhibited lower binding energy with the wild-type (WT) CPS1 protein (Thr1406) compared to the mutant-type (MT) protein (1406Asn) (Table 5). This differential binding suggests these compounds may preferentially interact with the WT protein.

Among these compounds, soyasaponin ag demonstrated the most pronounced difference in binding energy between WT and MT CPS1. The interaction between WT CPS1 and soyasaponin ag involved multiple binding forces, including hydrogen bonds, Van der Waals forces, alkyl interactions, π-alkyl interactions, and carbon–hydrogen bonds (Figure S3A). In contrast, the interaction between MT CPS1 and soyasaponin ag was primarily mediated by Van der Waals forces alone (Figure S3B).

Molecular docking analysis with vitamin D3 supported our cohort study findings. Vitamin D3 exhibited a lower binding energy to the mutant (MT) CPS1 protein (−9.9 kcal/mol) compared to the wild-type (WT) protein (−7.5 kcal/mol). In the WT CPS1 protein, van der Waals forces primarily mediated the interaction with vitamin D3, whereas, in the MT CPS1 protein, π-alkyl and alkyl interactions were more prominent, contributing to greater binding stability and reduced binding energy (Figure S4A,B). This aligns with our cohort study’s results, in which the adverse effect of the CPS1 rs1047891 risk allele on eGFR was observed only in individuals with low vitamin D intake (*p* = 0.0436; Table 4, Figure S4A,B). These findings collectively suggest that vitamin D intake may modify the impact of the CPS1 risk allele on renal function, potentially mitigating its negative effect (Figure 2C).

Despite identifying 19 bioactive compounds from plant-based foods with differential binding affinities to CPS1 WT versus MT protein in our molecular docking analyses, our cohort study did not demonstrate significant interactions between CPS1 rs1047891 and consumption of specific fruits or vegetables. While our molecular docking suggested potential interactions between CPS1 and various bioactive compounds from plant sources, these specific molecular interactions did not translate to detectable gene–diet interactions in our cohort study, unlike the clear interaction observed with vitamin D. This suggests that the protective effect of a PBD on eGFR observed at the PRS level may involve mechanisms beyond direct interactions with CPS1 or may reflect the cumulative effects of multiple dietary components acting through various pathways.

## 4. Discussion 

Declined eGFR represents a progressive loss of kidney function and is positively associated with urinary protein [31]. The present study confirmed this relationship, demonstrating that low eGFR was associated with urinary protein by 4.7 times. This indicates that decreased eGFR and increased urinary protein can serve as complementary indices of CKD. Importantly, reduced eGFR is linked not only to CKD but also to MetS and CVD.

A meta-analysis of GWAS data from European ancestry populations (61,286 controls and 5807 cases) identified 23 susceptible loci for reduced renal function [32]. In a follow-up study [32] with 22,982 replication samples, 13 loci were associated with renal function and CKD (*LASS2, GCKR, ALMS1, TFDP2, DAB2, SLC34A1, VEGFA, PRKAG2, PIP5K1B, ATXN2, DACH1, UBE2Q2*, and *SLC7A9*), while seven loci were involved in creatinine production and secretion (*CPS1, SLC22A2, TMEM60, WDR37, SLC6A13, WDR72*, and *BCAS3*). Our findings align with these previous studies, as the genetic variants with *p* < 1 × 10^−6^ related to low eGFR were primarily identified as being involved in creatinine production and secretion rather than kidney function and CKD directly. Furthermore, the genetic variants associated with low eGFR were identified as being associated with liver function, ATP production, myosin synthesis, and neuromuscular processes but not kidney function in the present study. The PRS from the selected genetic variants interacted with the PBD and coffee intake to affect low eGFR in the present study: high PBD and coffee intakes protected against low eGFR risk, especially in middle-aged and older people with a high PRS. These results can be applied to personalized nutrition.

In this study, we utilized the MDRD equation for eGFR estimation as our primary method for assessing kidney function. While the assessment of agreement between the CKD-EPI and MDRD equations was not a primary objective of our genetic association analysis, we verified the appropriateness of using MDRD in our Asian cohort. The verification showed strong concordance between the methods, with MDRD being highly predictive of CKD classification (OR = 0.115, 95% CI: 0.089–0.148). Given that the mean age of our study population was 53.7 years (SD: 8.0), we selected the MDRD equation as it has been shown to provide more stable eGFR estimates in aging adults without excessive CKD classification bias. This decision aligns with previous research in Chinese populations, which found that the MDRD exhibited a more gradual decline in eGFR with age than the CKD-EPI [19]. While the MDRD served the purposes of our genetic and lifestyle interaction analyses, we acknowledge that future studies investigating genetic determinants of kidney function in Asian populations should consider incorporating multiple eGFR estimation methods, including the CKD-EPI, to ensure robust phenotyping. This would be particularly important when evaluating gene–environment interactions across different age groups, as estimation methods may influence the detection and interpretation of genetic associations with kidney function.

Renal function is closely related to metabolic diseases [33]. As kidney function decreases, the metabolic pathways are altered to induce metabolic diseases such as MetS, CVD, gout, and cognitive dysfunction [6]. The changes could include altered blood volume and pressure, disturbed mineral metabolism, an activated renin-angiotensin system, oxidative stress, and increased inflammatory cytokines [34]. As a result, renal dysfunction contributes to increased insulin resistance to induce MetS and cardiac remodeling, which could progress to systolic and/or diastolic left ventricular failure [33]. The present study showed consistent results. Participants with a low eGFR had higher BMI and serum glucose and lipid concentrations than those with a high eGFR, indicating they were at a higher risk of MetS. Those with low eGFR had a higher prevalence of CVD and higher BUN and serum uric acid, AST, ALT, and hs-CRP concentrations. However, the serum albumin concentration was lower in participants with a low eGFR than in those with a high eGFR [33]. Therefore, renal function is closely connected with MetS, CVD, and gout and is linked to increased insulin resistance.

The relationship between eGFR and nutrient intake has been widely studied, focusing primarily on protein, sodium, and anti-inflammatory foods, but findings remain inconsistent. In this study, we found that high intakes of animal protein, essential amino acids, and fat were positively associated with low eGFR, while arginine intake, linked to the urea cycle, also showed a positive association. Previous studies have reported that high-fat intake (>35 En%) increases CKD risk (OR = 1.94, 95% CI: 1.39–2.71), while moderate protein intake (13–15 En%) may reduce risk (OR = 0.50, 95% CI: 0.35–0.72) in Mendelian randomization analyses using UK Biobank data [35]. However, longitudinal studies have suggested that high protein intake may accelerate eGFR decline in middle-aged adults [36]. While protein restriction (<10% total energy intake) may prevent renal decline, it can also lead to reduced lean body mass [37]. Given these findings, an optimal protein intake (~0.8 g/kg body weight) should be maintained to preserve renal function while preventing sarcopenia, particularly in older adults [38].

Although sodium intake has been linked to eGFR decline, findings remain inconsistent [39]. A high sodium intake was associated with eGFR decline in a longitudinal study [40], while a meta-analysis of eight randomized trials found that salt reduction lowered blood pressure and proteinuria in CKD patients [41]. In the present study, sodium and potassium intakes were lower in the Low-GFR group, but no association with low eGFR was found, possibly due to dietary sodium restriction as part of clinical recommendations. However, the potassium-to-sodium ratio was positively associated with low eGFR, consistent with prior cross-sectional findings [40]. These results suggest that sodium reduction may help maintain kidney function, though further longitudinal studies are needed to confirm this relationship.

Genetic variants influence renal function and creatinine metabolism, with multiple loci identified in European and Asian populations [42]. While many eGFR-associated variants are pleiotropic and shared across populations, some are unique to specific ancestries. Our study identified variants in *SHROOM3* and *SLC34A1*, previously associated with CKD risk, and others, including *CPS1, WDR37*, and *WDR72*, linked to creatinine metabolism and secretion. Given that *CPS1* plays a central role in nitrogen metabolism and ammonia detoxification via the urea cycle, genetic variation in *CPS1* could affect renal function through altered amino acid metabolism and nitrogen balance.

In this study, we found that the PRS of the 6-SNP model for low eGFR interacted with PBD and coffee intake but not with WSD, high protein, or sodium intake. The impact of high PRS was lower in participants with a high PBD intake, though a PBD itself was not associated with renal function in the overall population. This aligns with previous studies in US and Chinese populations, where higher adherence to a PBD and vegetarian diets was inversely associated with low eGFR in middle-aged adults [43,44]. These findings suggest that a PBD may provide a protective effect on eGFR, particularly in individuals with genetic susceptibility to renal dysfunction.

The protective effects of polyphenols from plant-based foods on renal function likely involve multiple mechanisms. Polyphenols are known to exert antioxidant and anti-inflammatory effects, which may protect against oxidative stress and inflammation-induced kidney damage [45]. Additionally, polyphenols may modulate signaling pathways involved in renal cell survival and function. Our molecular docking analyses suggest a potential direct interaction between certain polyphenolic compounds and CPS1, which could influence nitrogen metabolism and subsequently impact kidney function. However, the absence of specific interactions between CPS1 and the majority of plant-derived bioactive compounds in our molecular docking studies, coupled with the observed protective effect of a PBD on renal function in our cohort, suggests that the beneficial effects of polyphenols may be mediated through broader metabolic pathways rather than direct modulation of CPS1 activity alone.

Additionally, our results support a beneficial effect of coffee intake on kidney function, consistent with prior research showing that drinking more than daily one cup of coffee is associated with higher eGFR and lower albuminuria in Mendelian randomization analyses [46]. A meta-analysis of 12 studies also found that coffee consumption was linked to a lower risk of CKD (RR = 0.86, 95% CI: 0.76–0.97) and albuminuria (OR = 0.81, 95% CI: 0.68–0.97) [47]. In the present study, coffee intake was inversely associated with renal dysfunction, and the PRS-low eGFR association was weaker in those with high coffee intake, indicating a gene–diet interaction influencing renal function. These findings suggest that coffee consumption may help protect against renal dysfunction, particularly in individuals at higher genetic risk.

Our study also revealed a significant interaction between *CPS1* rs1047891 and vitamin D intake, where low vitamin D intake was associated with decreased eGFR in risk allele carriers, while high vitamin D intake mitigated this effect. Prior studies have shown that vitamin D plays a key role in kidney health by regulating calcium-phosphate homeostasis, immune function, and oxidative stress [48,49]. Additionally, CPS1 is a key enzyme in nitrogen metabolism, and mutations in CPS1 have been linked to urea cycle disorders, hyperammonemia, and impaired metabolic function [50]. To further explore this interaction, molecular docking analysis was performed using AutoDock Vina, demonstrating that vitamin D3 exhibited a lower binding energy to CPS1 mutant-type (−9.9 kcal/mol) than wild-type (−7.5 kcal/mol). This finding aligns with our cohort results showing that vitamin D intake modifies the effect of the CPS1 risk allele on renal function and provides a potential molecular mechanism for this gene–nutrient interaction.

It is worth noting that while our molecular docking analyses identified differential binding patterns between vitamin D3 and *CPS1* variants that correspond with our cohort findings, similar correlations were not observed for bioactive compounds from plant-based foods. Despite identifying 19 compounds from vegetables and fruits with different binding energies to wild-type versus mutant *CPS1*, our cohort study did not demonstrate significant interactions between specific fruits or vegetables and *CPS1* rs1047891. This suggests that the protective effect of a PBD on eGFR observed at the PRS level may involve complex mechanisms beyond direct interactions with *CPS1*, potentially including synergistic effects of multiple polyphenols acting through various pathways. This complexity highlights the limitations of in silico analyses in predicting functional outcomes and emphasizes the importance of validation through biological assays and human studies.

The major limitation of this study was that the cause-and-effect relationship could not be established because of its case–control design. Second, renal function was estimated using eGFR calculated with the MDRD equation, which, while suitable for our study population, may have limitations in assessing acute kidney injury and could differ from other eGFR estimation methods, such as CKD-EPI. Third, alcohol intake was analyzed as total daily consumption, without distinguishing the potential differential effects of beer, wine, and spirits on renal function. Finally, dietary intake was assessed with an SQFFQ for the previous six months. However, since the SQFFQ (106 food items) was validated against 3-day food records collected across four seasons, it is likely to provide a reliable estimate of the usual intake in this population.

## 5. Conclusions

The present study reveals a significant association between renal dysfunction and insulin resistance and dietary factors like WSD, with fast-food intake exacerbating this relationship. Genetic variants linked to renal function also showed connections to metabolic pathways in the liver and skeletal muscle. Notably, our 6-SNP polygenic risk score demonstrated a 1.782-fold increased risk for renal dysfunction and interacted with a PBD and coffee consumption. These findings suggest that dietary modifications—particularly a PBD and moderate coffee intake—may mitigate genetic risk for renal dysfunction in middle-aged and elderly individuals. These results support integrating genetic risk assessments into personalized nutritional recommendations for kidney health, potentially enhancing CKD prevention strategies in at-risk populations. Future research should validate these findings through longitudinal studies and explore the underlying mechanisms through functional studies of relevant genetic variants and bioactive compounds.

## Figures and Tables

**Table 1:** Parameters related to metabolic diseases according to the estimated glomerular filtration rates (eGFR) ^1^.

	High-GFR (*n* = 51,084)	Low-GFR (*n* = 7617)	Adjusted OR and 95% CI
Age (years) ^2^	53.3 ± 0.03	56.5 ± 0.08 ***	2.156 (1.975–2.354)
Gender (male, N %)	16,105 (33.6)	3332 (43.7) ^+++^	0.701 (0.649–0.756)
Education <High school	7268 (19.6)	1223 (21.2)	1
High school	27,133 (73.2)	4172 (72.2)	1.130 (0.978–1.306)
>High school	2687 (7.24)	412 (7.09)	1.194 (0.912–1.562)
MetS (N, %) ^3^	6998 (13.7)	2148 (28.2) ^+++^	1.991 (1.781–2.225)
CVD (N, %) ^4^	1890 (3.72)	799 (10.5) ^+++^	1.920 (1.625–2.268)
BMI (kg/m^2^) ^5^	23.9 ± 0.03	24.3 ± 0.05 ***	1.374 (1.244–1.518)
Waist Cir. (cm) ^6^	80.6 ± 0.03	81.7 ± 0.18 ***	1.304 (1.168–1.456)
Serum glucose (mg/dL) ^7^	95.1 ± 0.09	97.6 ± 0.48 **	1.853 (1.632–2.105)
Blood HbA1c (%) ^8^	5.71 ± 0.004	5.96 ± 0.026 ***	1.598 (1.339–1.908)
Serum HDL (mg/dL) ^9^	53.9 ± 0.06	51.1 ± 0.31 ***	0.662 (0.598–0.733)
Serum LDL (mg/dL) ^10^	118 ± 0.15	120 ± 0.39 ***	1.277 (1.143–1.427)
Serum TG (mg/dL) ^11^	124 ± 0.39	130 ± 1.00 ***	1.340 (1.210–1.483)
SBP (mmHg) ^12^	122 ± 0.07	122 ± 0.17	1.142 (1.032–1.263)
DBP (mmHg) ^13^	75.7 ± 0.04	75.8 ± 0.11	1.103 (0.943–1.289)
Hypertension (N, %)	2814 (5.51)	1150 (15.1) ^+++^	2.089 (1.887–2.312)
Insulin resistance (N, %)	3918 (7.67)	975 (12.8)***	1.473 (1.271–1.707)
Serum CRP (mg/dL) ^14^	0.137 ± 0.002	0.154 ± 0.005 **	1.714 (1.254–2.344)
Serum creatinine (mg/dL)	0.79 ± 0.001	1.24 ± 0.003	
Serum uric acid (mg/dL) ^15^	4.60 ± 0.005	5.24 ± 0.01 ***	4.531 (4.036–5.087)
Blood urinary nitrogen (mg/dL) ^16^	14.4 ± 0.02	18.2 ± 0.05	3.247 (2.935–3.591)
Albumin (mg/dL) ^17^	4.62 ± 0.001	4.59 ± 0.006 ***	0.753 (0.683–0.831)
AST (U/L) ^18^	23.6 ± 0.11	24.7 ± 0.30 ***	1.189 (0.957–1.477)
ALT (U/L) ^19^	22.3 ± 0.11	23.0 ± 0.29 *	1.170 (1.010–1.355)
Total bilirubin ^20^	0.73 ± 0.002	0.74 ± 0.005 *	2.432 (2.306–2.565)
Urinary protein ^21^	1.09 ± 0.002	1.33 ± 0.01 ***	4.653 (3.919–5.524)

^1^ The MDRD Study’s equation using serum creatinine concentrations and the High-GFR and Low-GFR groups were defined as > and ≤60 mL/min/1.73 m^2^ of eGFR. OR, odds ratio; CI, confidence intervals. The cutoff points for moderate exercise in the logistic regression were as follows: ^2^ <55 years old. ^3^ Metabolic syndrome (MetS) criteria. ^4^ Cardiovascular diseases (CVDs). ^5^ <25 kg/m^2^ for body mass index (BMI). ^6^ <90 cm for men and 85 cm for women waist circumferences (Cir.). ^7^ <126 mL/dL fasting serum glucose plus diabetic drug intake. ^8^ <6.5% hbA1c plus diabetic drug intake. ^9^ >40 mg/dL for men and 50 mg/dL for women serum HDL cholesterol. ^10^ <160 mg/dL serum LDL cholesterol. ^11^ <150 mg/dL serum triglyceride (TG). ^12^ <140 mmHg systolic blood pressure (SBP). ^13^ <90 mmHg diastolic blood pressure (DBP) plus hypertension medication. ^14^ <0.5 mg/dL serum high-sensitivity C-reactive protein (hs-CRP). ^15^ <7 mg/dL for men and 6 mg/dL for women serum uric acid. ^16^ <24 mg/dL blood urinary nitrogen (BUN). ^17^ <4.8 mg/dL serum albumin concentrations. ^18^ <40 U/L Aspartate aminotransferase (AST). ^19^ <35 U/L alanine aminotransferase (ALT). ^20^ <1.2 mg/dL serum total bilirubin concentrations. ^21^ Negative urinary protein. Covariates: sex, age, residence area, lean body mass, education, income, married status, lean body mass, energy and sodium intake, smoking, alcohol drinking, and exercise. * Significant differences by GFR at *p* < 0.05, ** *p* < 0.01, and *** *p* < 0.001. ^+++^ Significantly different from the control group in the χ^2^ test at *p* < 0.001.

**Table 2:** Characteristics of the ten genetic variants selected for estimated glomerular filtration rates.

CHR ^1^	SNP ^2^	Position	Mi ^3^	Ma ^4^	OR ^5^	SE ^6^	Adjusted *p*-Value ^7^	Adjusted *p*-Value ^8^	MAF ^9^	*p*-Value for HWE ^10^	Gene Names	Location
2	rs1047891 (Thr1406Asn)	211540507	A	C	1.172	0.0241	4.61 × 10^−11^	2.98 × 10^−5^	0.179	0.1156	*CPS1*	Missense
2	rs3770636	170202833	G	T	0.881	0.0245	2.3 × 10^−7^	0.00692	0.200	0.0692	*LRP2*	Intron
4	rs5020545	77414988	T	C	1.149	0.0247	1.83 × 10^−8^	0.00724	0.171	0.6204	*SHROOM3*	Intron
5	rs3812036	176813404	T	C	1.122	0.0227	3.97 × 10^−7^	0.0003224	0.219	0.7723	*SLC34A1*	Intron
6	rs4715517	54973761	A	C	1.283	0.0396	2.88 × 10^−10^	0.0002845	0.054	0.3498	*HCRTR2*	5′-UTR
6	rs140652052	33547810	C	A	1.259	0.0464	6.95 × 10^−7^	0.009824	0.038	0.8193	*BAK1*	Intron
7	rs139132767	116726021	G	A	1.579	0.0616	1.22 × 10^−13^	0.008934	0.018	0.2941	*ST7*	Intron
12	rs141574969	111319366	G	A	1.142	0.0257	2.15 × 10^−7^	0.007747	0.159	0.1384	*CCDC63*	Intron
15	rs1031755	53951435	C	A	0.8967	0.0198	3.49 × 10^−8^	0.00043	0.403	0.4817	*WDR72*	Intron
22	rs6001939	40892794	T	C	0.9098	0.0213	9.21 × 10^−7^	0.001771	0.293	0.2773	*MRTFA*	Intron

^1^ Chromosome. ^2^ Single-nucleotide polymorphism. ^3^ Minor allele. ^4^ Major allele. ^5^ Adjusted odds ratio for renal dysfunction in a hospital-based cohort. ^6^ Standard error. ^7^ p-Value for the OR in the urban hospital-based cohort (case: *n* = 1361, control: *n* = 12,237). ^8^ *p*-Value for OR in the Ansan/Ansung + Nong-chon cohort (case: *n* = 7617, control: *n* = 51,084). ^9^ Minor allele frequency. ^10^ Hardy–Weinberg equilibrium. Covariates: sex, age, residence area, lean body mass, education, income, married status, lean body mass, energy and sodium intake, smoking, alcohol drinking, and exercise.

**Table 3:** Pathways related to genetic variants influencing renal function determined by estimated glomerular filtration rates.

Gene Set	No. Genes	Beta	Beta SD	SE	*p*-Value
GO BP: Protein autoprocessing	28	0.59988	0.02312	0.15852	7.73 × 10^−5^
GO BP: Regulation of receptor recycling	22	0.66108	0.02259	0.17705	9.46 × 10^−5^
Acevedo liver cancer dn	516	0.1403	0.02291	0.03774	0.0001007
GO BERT: Core oligodendrocyte differentiation	40	0.48444	0.02231	0.14096	0.0002951
GO CC: Neuronal ribonucleoprotein granule	3	1.6359	0.02065	0.48121	0.0003383
Biocarta LDL pathway	6	1.2646	0.02257	0.37738	0.0004037
Bhat esr1 targets via akt1 up	273	0.17603	0.02104	0.05297	0.0004456
GO CC: Proton transporting atp synthase complex catalytic core f1	6	1.0336	0.01845	0.31139	0.0004519
GO BP: Negative regulation of receptor recycling	5	1.0453	0.01703	0.31814	0.0005096
GO BP: Negative regulation of myosin light chain phosphatase activity	5	1.4803	0.02412	0.4553	0.0005756

SD, standard deviation; SE, standard error; GO, gene ontology; CC, cellular compartment; BP, biological process.

**Table 4:** Adjusted odds ratios and 95% confidence intervals for renal dysfunction risk by polygenetic risk scores (PRS) of the 6-SNP model or *CPS1* rs1047891 after covariate adjustments according to lifestyle patterns.

PRS 6	Low-PRS (*n* = 17,680)	Medium-PRS (*n* = 26,672)	High-PRS (*n* = 9476)	PRS Interaction *p*-Value
Low-ABD ^1^ High-ABD	1 1	1.247 (1.151–1.351) 1.396 (1.250–1.558)	1.534 (1.390–1.693) 1.665 (1.452–1.910)	0.2522
Low-PBD ^1^ High-PBD	1 1	1.371 (1.266–1.484) 1.162 (1.039–1.299)	1.628 (1.475–1.797) 1.457 (1.275–1.686)	0.0215
Low-WSD ^1^ High-WSD	1 1	1.305 (1.199–1.420) 1.285 (1.162–1.421)	1.555 (1.400–1.728) 1.611 (1.424–1.822)	0.9430
Low-RMD ^1^ High-RMD	1 1	1.331 (1.230–1.440) 1.232 (1.100–1.379)	1.592 (1.444–1.755) 1.558 (1.354–1.792)	0.0756
Low-Sodium ^2^ High-Sodium	1 1	1.301 (1.179–1.435) 1.298 (1.191–1.414)	1.501 (1.328–1.697) 1.649 (1.484–1.833)	0.8257
Low-Alcohol ^3^ High-Alcohol	1 1	1.226 (1.124–1.336) 1.391 (1.261–1.534)	1.526 (1.376–1.694) 1.639 (1.445–1.859)	0.2312
Non-smoker Former + Current smokers	1 1	1.284 (1.187–1.388) 1.338 (1.192–1.502)	1.620 (1.472–1.784) 1.522 (1.318–1.757)	0.4421
Low Exercise Regular Exercise ^4^	1 1	1.140 (1.033–1.259) 1.419 (1.304–1.545)	1.431 (1.267–1.615) 1.709 (1.538–1.898)	0.0596
Low-Coffee ^5^ High-Coffee	1 1	1.311 (1.220–1.409) 1.248 (1.078–1.445)	1.655 (1.513–1.809) 1.344 (1.121–1.611)	0.0092
Low-Tea ^6^ High-Tea	1 1	1.307 (1.212–1.409) 1.272 (1.119–1.446)	1.609 (1.466–1.765) 1.496 (1.275–1.754)	0.2290
Low-Vitamin D ^7^ High-Vitamin D	1 1	1.327 (1.169–1.505) 1.280 (1.191–1.376)	1.574 (1.440–1.721) 1.521 (1.300–1.780)	0.2066
*CPS1* rs1047891	Major	Heterozygote	Minor	
Low-Vitamin D ^7^ High-Vitamin D	1 1	1.235 (1.099–1.388) 1.176 (1.099–1.257)	1.393 (1.190–1.632) 1.124 (0.833–1.517)	0.0436

The cutoffs of the logistic regression analysis were as follows: ^1^ <66th percentiles; ^2^ 2 g/day sodium intake; ^3^ 140 g/week alcohol intake; ^4^ moderate activity for 150 min/week; ^5^ 3 g/day coffee intake; ^6^ <45 g/day tea intake; and ^7^ <2.53 μg/day vitamin D intake (33rd percentile). The High-GFR and Low-GFR groups were defined as ≥ and <60 mL/min/1.73 m^2^ of eGFR. Low-PRS (0–4), Medium-PRS (5–6), and High-PRS (>6) in the 6-SNP model. Covariates: sex, age, residence area, lean body mass, education, income, married status, lean body mass, energy and sodium intake, smoking, alcohol drinking, and exercise. ABD, Asian balanced diet; PBD, plant-based diet; WSD, Western-style diet; RMD, rice—main diet.

**Table 5:** Bioactive compounds lower intermolecular binding energy with CPS1 (Thr1406Asn) wild type (WT) than the mutated type (MT).

Food Components	Foods	WT	MT
		Docking Energy, ΔG (kcal mol^−1^)	
Cichoriin	Chicory	−10.1	−8.6
Malvidin 3-alpha-L-galactoside	Blue berry	−10.1	−8.6
Glyceollin II	Soybeans	−10.6	−8.7
Stigmasteryl glucoside	Soybean oil	−10.2	−8.5
alpha-Carotene	Carrot	−10.9	−8.6
(5R,5’R,6S,8′R)-Luteochrome	Sweet potato	−10.2	−7.3
Tuberoside B	Allium tuberosum	−10.6	−8.7
Cycloartanyl ferulate	Rice bran oil	−10.3	−8.6
delta-Carotene	Carrot tomatoes	−10.5	−7.7
19’-Hexanoyloxymytiloxanthin	Mussel	−10.2	−8
(R)-Hispaglabridin A	Licorice	−10.2	−8.6
5,6,7,8-Tetrahydroxy-3’,4’-dimethoxyflavone	Seville orange	−10.6	−8.7
(3S,3’S,all-E)-Zeaxanthin	Shrimp	−10.9	−8.3
(E)-4-(3,7-Dimethyl-2,6-octadienyl)-1,3,5-trihydroxyxanthone	Garcinia livingstonei	−10.1	−8.5
28-Hydroxymangiferonic acid	Mango	−10.5	−8.5
Soyasaponin ag	Soybeans, pulses	−11.3	−8.2
(S)-Nerolidol 3-O-[a-L-Rhamnopyranosyl-(1->4)-a-L-rhamnopyranosyl-(1->2)-[4-(4-hydroxy-3-methoxycinnamoyl)-(E)-a-L-rhamnopyranosyl-(1->6)]-b-D-glucopyranoside]	Eriobotrya japonica	−10.5	−8.6
3alpha-12-Ursene-3,24-diol	Boswellia serrata	−10.6	−8.5
Hovenidulcioside B2	Hovenia dulcis	−10.5	−8.6
Vitamin D3	Egg yolk, liver, tuna	−7.6	−9.9

## Data Availability

The data were deposited in Korean biobank (Osong, Korea) and provided for the research.

## Supplementary Materials

The following supporting information can be downloaded at: https://www.mdpi.com/article/10.3390/nu17050916/s1, Table S1: Nutrient intake according to estimated glomerular filtration rates (eGFR); Table S2: Generalized multifactor dimensionality reduction (GMDR) results of multi-locus interaction with genes related to glomerular filtration rate risk; Figure S1: A flow chart to generate the polygenic risk score (PRS) linked to duodenal ulcers by SNP–SNP interaction and its interaction with lifestyle factors; Figure S2: Distribution of genetic variants associated with duodenal ulcers based on the genome-wide association study (GWAS); Figure S3: Molecular docking of CPS1 WT protein (Thr1406) (A) and MT (1406Asn) (B) with soyasaponin ag; Figure S4: Molecular docking of CPS1 WT protein (Thr1406) (A) and MT (1406Asn) (B) with vitamin D3.
