# Supplementary material for: Gene–Lifestyle Interactions in Renal Dysfunction: Polygenic Risk Modulation via Plant-Based Diets, Coffee Intake, and Bioactive Compound Interactions
Source: Nutrients. 2025 Mar 6;17(5):916. doi: 10.3390/nu17050916 (PMC11901526; doi:10.3390/nu17050916)
Supplement: Supplementary file 1 [file nutrients-17-00916-s001.zip › nutrients-3497690-supplementary.pdf]

Table S1. Nutrient intake according to estimated glomerular filtration rates (eGFR)

	High-GFR (n=47919)	Low-GFR (n=7617)	Adjusted ORS and 95% CI
Energy (EER %)	96.3±0.14[1]	93.7±0.73[**]	0.995 (0.944-1.048)[2]
CHO (70 En%)	71.5±0.03	71.4±0.08	0.951 (0.899-1.006)
Protein (15 En%)	13.4±0.01	13.5±0.03	1.030 (0.978-1.085)
Animal protein (En%)[1]	2.17±0.01	2.31±0.03[***]	1.147 (1.077-1.222)
Essential amino acid (mg/d)[1]	3323±3.34	3382±18.3[**]	1.174 (1.014-1.359)
Non-essential amino acid (mg/d)[1]	5541±5.24	5521±13.3	1.050 (0.962-1.145)
Arginine (mg/d)[3]	417±0.61	422±1.56[**]	1.119 (1.037-1.209)
Glutamate (mg/d)[3]	2135±2.82	2117±7.24[*]	0.963 (0.900-1.030)
Cysteine (mg/d)[3]	89.9±0.11	89.3±0.29[*]	0.921 (0.859-0.986)
Tryptophan (mg/d)[3]	94.2±0.13	93.4±0.33[*]	1.004 (0.935-1.079)
Aspartate (mg/d)[3]	724±0.77	727±1.97	1.052 (0.967-1.143)
Leucine (mg/d)[3]	748±1.56	746±2.92	1.051 (0.926-1.194)
Isoleucine (mg/d)[3]	378±0.96	381±1.79[*]	1.159 (1.019-1.319)
Valine (mg/d)[3]	473±1.12	473±2.08	1.060 (0.928-1.210)
Fat (15 En%)	14.0±0.02	14.2±0.06[*]	1.064 (1.006-1.125)
Saturated fat (4.7 En%)	4.49±0.12	4.59±0.30[***]	1.102 (1.039-1.169)
MUFA (6.0 En%)	5.61±0.13	5.69±0.03[*]	1.056 (0.993-1.123)
PUFA (2.5 En%)	3.15±0.11	3.21±0.27[*]	1.023 (0.963-1.087)
Calcium (700 mg/d)	451.1±2.43	449±4.54	0.982 (0.883-1.093)
Sodium (Na; 2 g/d)	2.44±0.006	2.34±0.03[**]	1.043 (0.959-1.134)
Potassium (K;2 g/d)	2.23±0.004	2.17±0.02[**]	0.959 (0.882-1.044)
Na/K[1] (0.78)	1.04±0.002	1.07±0.010[**]	1.142 (1.017-1.282)
V-D (2.53 μg/d)	6.60±0.05	6.60±0.10	0.977 (0.882-1.082)
V-C (100 mg/d)	111±0.65	112±1.22	1.038 (0.942-1.144)
DII[1]	-19.9±0.06	-19.8±0.14	1.052 (0.983-1.125)
Flavonoids (mg/d)[3]	41.1±0.34	41.4±0.64	1.046 (0.955-1.145)
ABD (N, High%)	16840 (33.3)	2729 (33.4)	1.044 (0.981-1.111)
PBD (N, High%)	16826 (33.3)	2750 (33.7)	1.031 (0.972-1.094)
WSD (N, High%)	20413 (40.4)	3239 (42.5)[+++]	1.122 (1.058-1.189)
RMD (N, High%)	16973 (33.6)	2598 (31.8)[++]	1.043 (0.986-1.104)
Former smoker +smoker (N, Yes%)	12527 (26.1)	2380 (31.2)[+++]	1.000 (0.861-1.161)
Alcohol (g/week)[4]	112±1.38	88.0±7.54[**]	0.896 (0.846-0.949)
Coffee (g/d)[5]	3.67±0.01	3.53±0.06[*]	0.849 (0.741-0.973)
Tea (g/d)[6]	43.2±0.37	46.1±2.02	1.086 (0.983-1.199)

Exercise (N, Yes%)	26,116 (54.5)	4463 (58.6)++	1.069 (0.967-1.181)

[1]Values represent adjusted mean and standard errors for continuous variables and the number and percentage for categorical variables. [2]Values represent adjusted odd ratios (ORs) and 95% confidence intervals (CI). Covariates included age, sex, education, income, energy intake (percentage of estimated energy requirement), residence areas, daily activity, alcohol intake, and smoking status. [3]The cutoff points of the reference for logistic regression were <66th percentile, [4]140 g/week alcohol intake; [5]3 g/day coffee intake, and [6]45 g/day tea intake. The High-GFR and Low-GFR groups were defined as > and ≤ 60 mL/min/1.73 m^2 of eGFR. CHO, carbohydrates; MUFA, monounsaturated fatty acids; PUFA, polyunsaturated fatty acids; DII, dietary inflammatory index; ABD, Asian balanced diet; PBD, plant-based diet; WSD, Western-style diet; RMD, rice-main diet.

* Significant differences by GFR at P<0.05, ** at P<0.01, *** P<0.001.

++ Significantly different from the control group in χ^2 test at P<0.01, +++ at P<0.001.

Table S2. Generalized multifactor dimensionality reduction (GMDR) results of multi-locus interaction with genes related to glomerular filtration rate risk

Model	TRBA	TEBA	P value	CVC	TRBA	TEBA	P value	CVC
*CCDC63*_rs141574969	0.5188	0.5126	0.001	7	0.5188	0.5126	0.001	7
*SHROOM3*_rs5020545, *HCRTR2*_rs4715517	0.5255	0.5209	0.001	8	0.5255	0.5209	0.001	8
*BAK1*_rs140652052 plus model 2	0.5299	0.5172	0.0107	4	0.5299	0.5172	0.0107	4
*LRP2*_rs3770636, *CPS1*_rs1047891, *SHROOM3*_rs5020545, *CCDC63*_rs141574969	0.535	0.5184	0.001	3	0.535	0.5184	0.001	3
*CPS1*_rs1047891, *SHROOM3*_rs5020545 *SLC34A1*_rs3812036, *CCDC63*_rs141574969, *WDR72*_rs491567	0.5434	0.5281	0.001	9	0.5434	0.5281	0.001	9
*SLC34A1*_rs3812036, *MRTFA*_rs6001939, plus model 4	0.5559	0.5259	0.001	10	0.5559	0.5259	0.001	10
*WDR72*_rs491567 plus model 6	0.5749	0.5176	0.0107	10	0.5749	0.5176	0.0107	10
*HCRTR2*_rs4715517 plus model 7	0.59	0.5167	0.001	10	0.59	0.5167	0.001	10
*BAK1*_rs140652052 plus model 8	0.6045	0.5205	0.001	10	0.6045	0.5205	0.001	10
*ST7*_rs139132767 plus model 9	0.6148	0.5208	0.001	10	0.6148	0.5208	0.001	10

TRBA, training balanced accuracy. P value: the sign test of testing balanced accuracy (TEBA); CVC, Cross-validation consistency.

Figure S1. Flow chart to generate the polygenic risk score (PRS) linked to duodenal ulcers by SNP-SNP interaction and its interaction with lifestyle factors

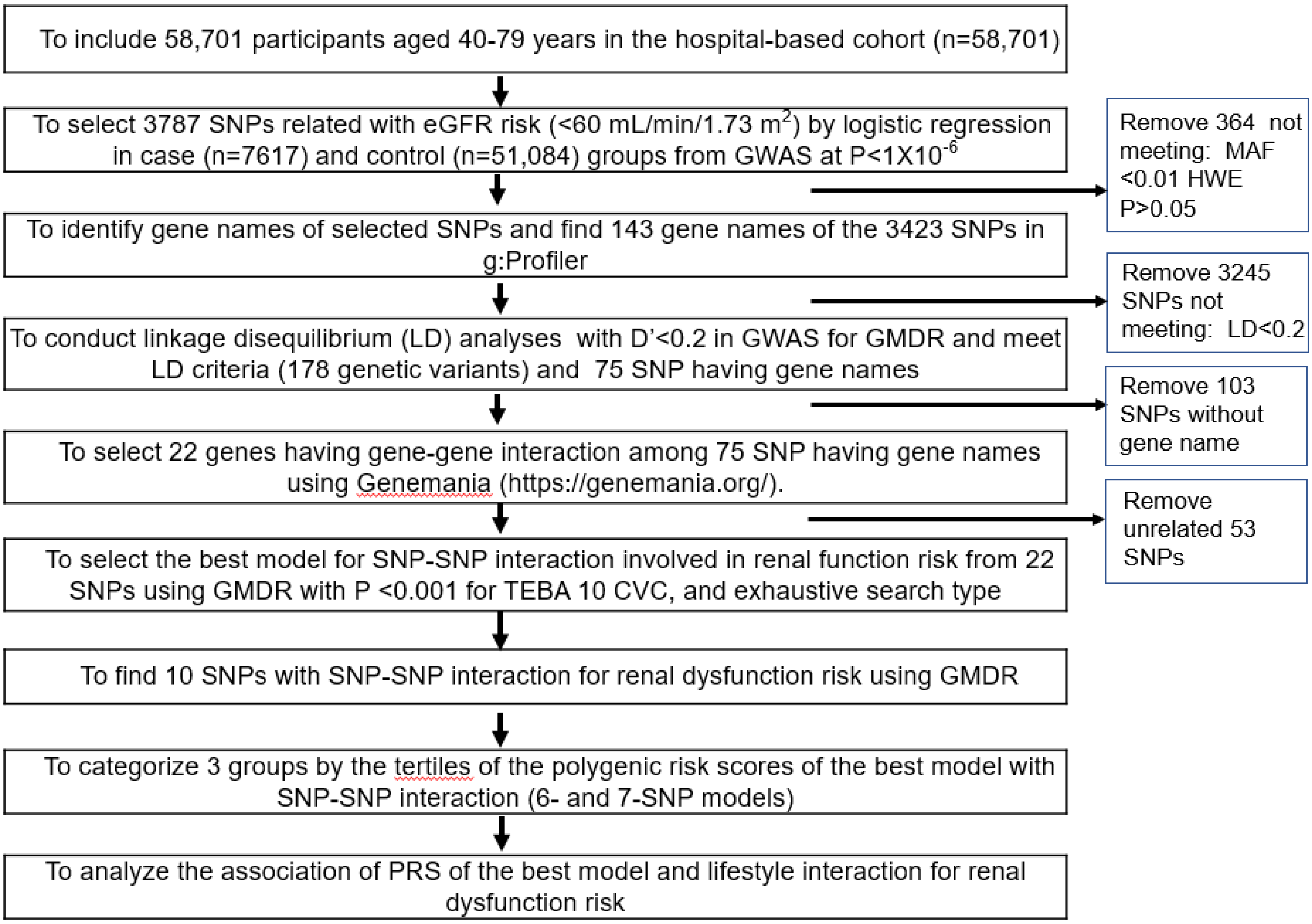

SNP: Single nucleotide polymorphisms

Figure S2. Distribution of genetic variants associated with duodenal ulcers based on the genome-wide association study (GWAS).

A. Manhattan plot of the *p*-value of genetic variants for duodenal ulcer risk.

B. Q–Q plot of observed and expected *p*-values for duodenal ulcer risk.

Q-Q: Quantile-quantile

A

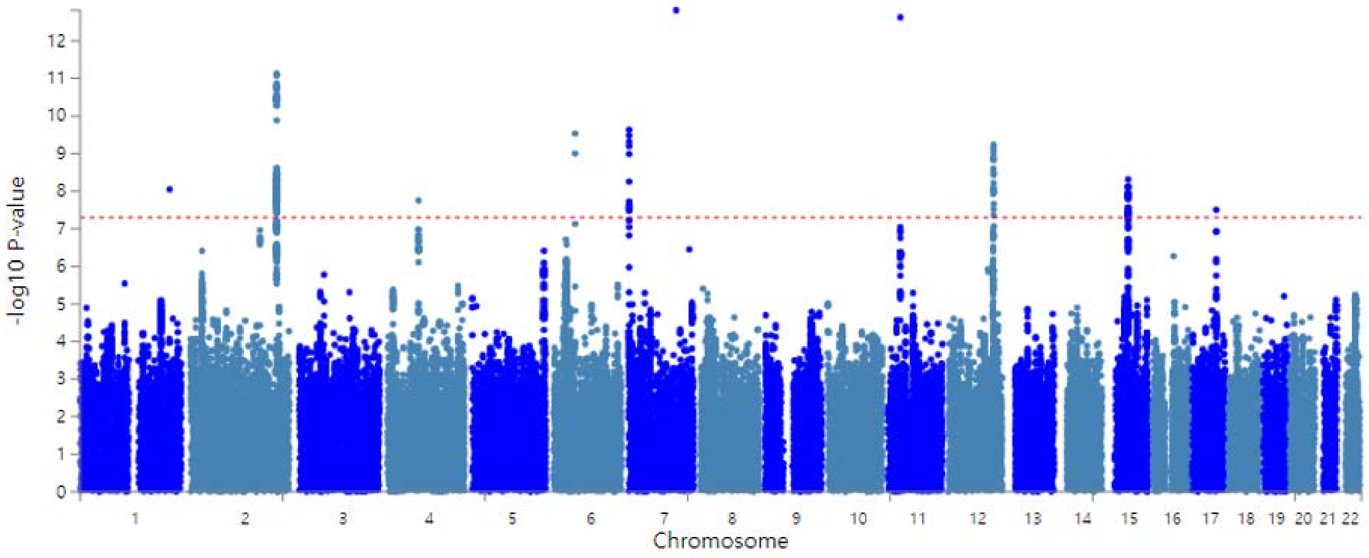

B

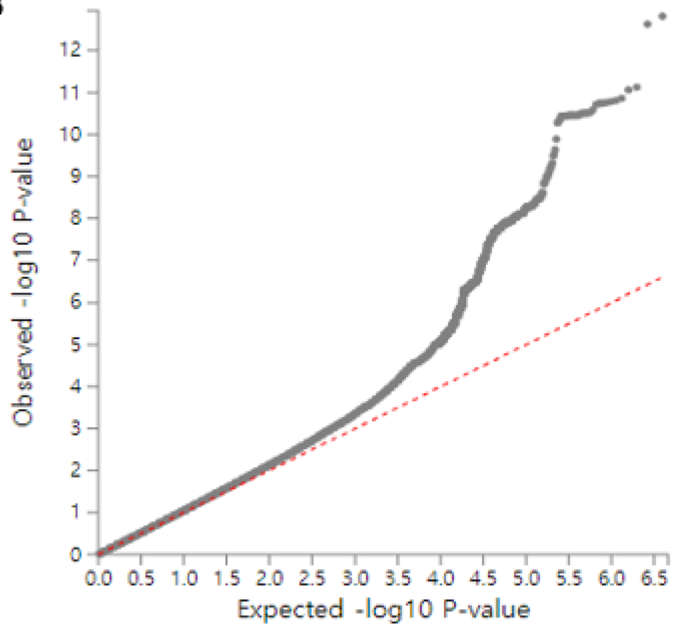

Figure S3. Molecular docking of CPS1 WT protein (Thr1406)(A) and MT (1406Asn)(B) with soyasaponin ag. Molecular docking with the bioactive compound and carbamoyl phosphate synthetase-1 (CPS1) wild-type protein (WT, Thr1406). (a) Diagrammatic representation of the compound (balls and stick model) binding with CPS1, (b) 2D depiction of CPS1 interacting with the compound and the nature of forces involved in stabilizing CPS1–soyasaponin ag-complex, and (c) Binding of the compound at the central cavity of CPS1.

A

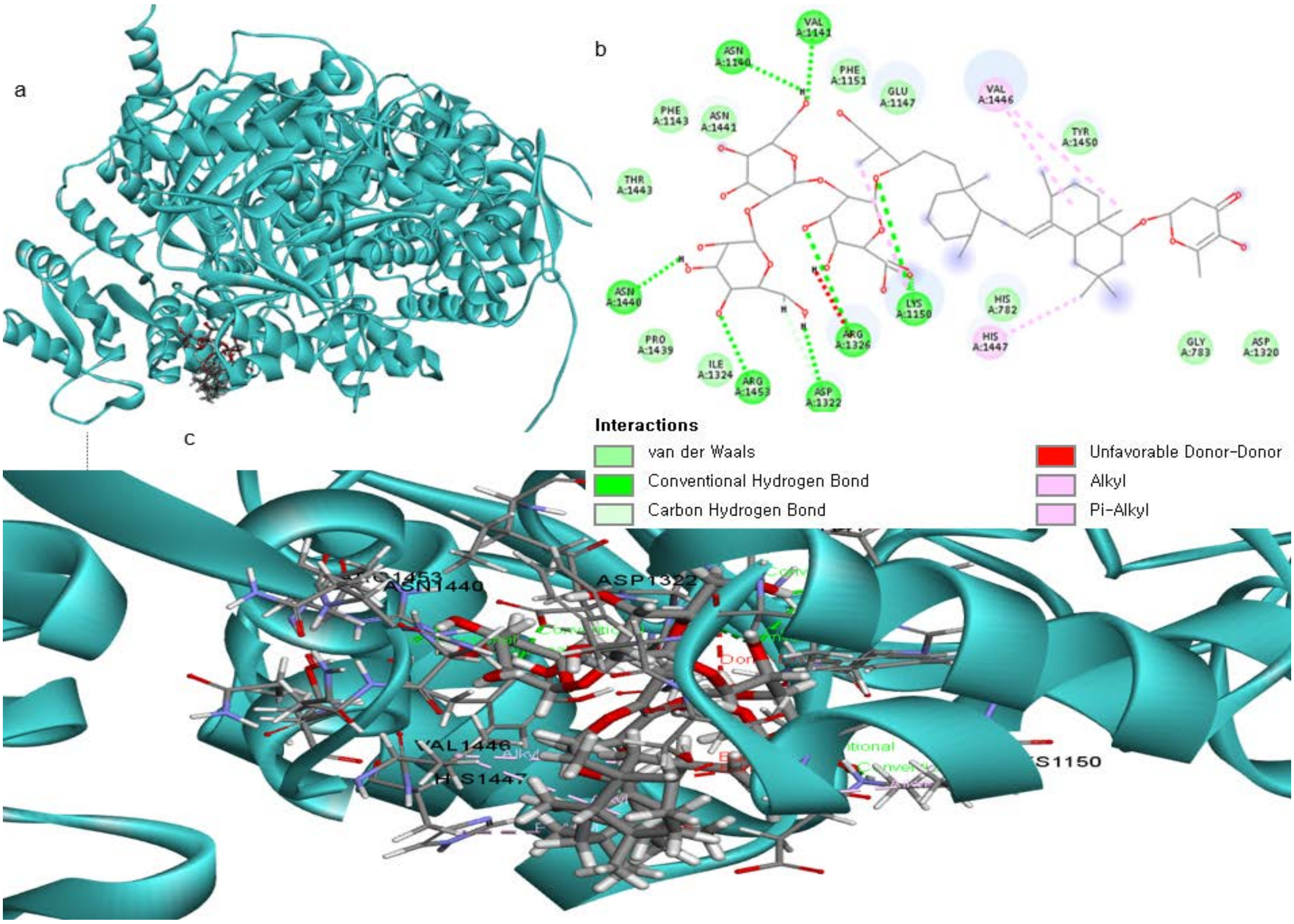

B

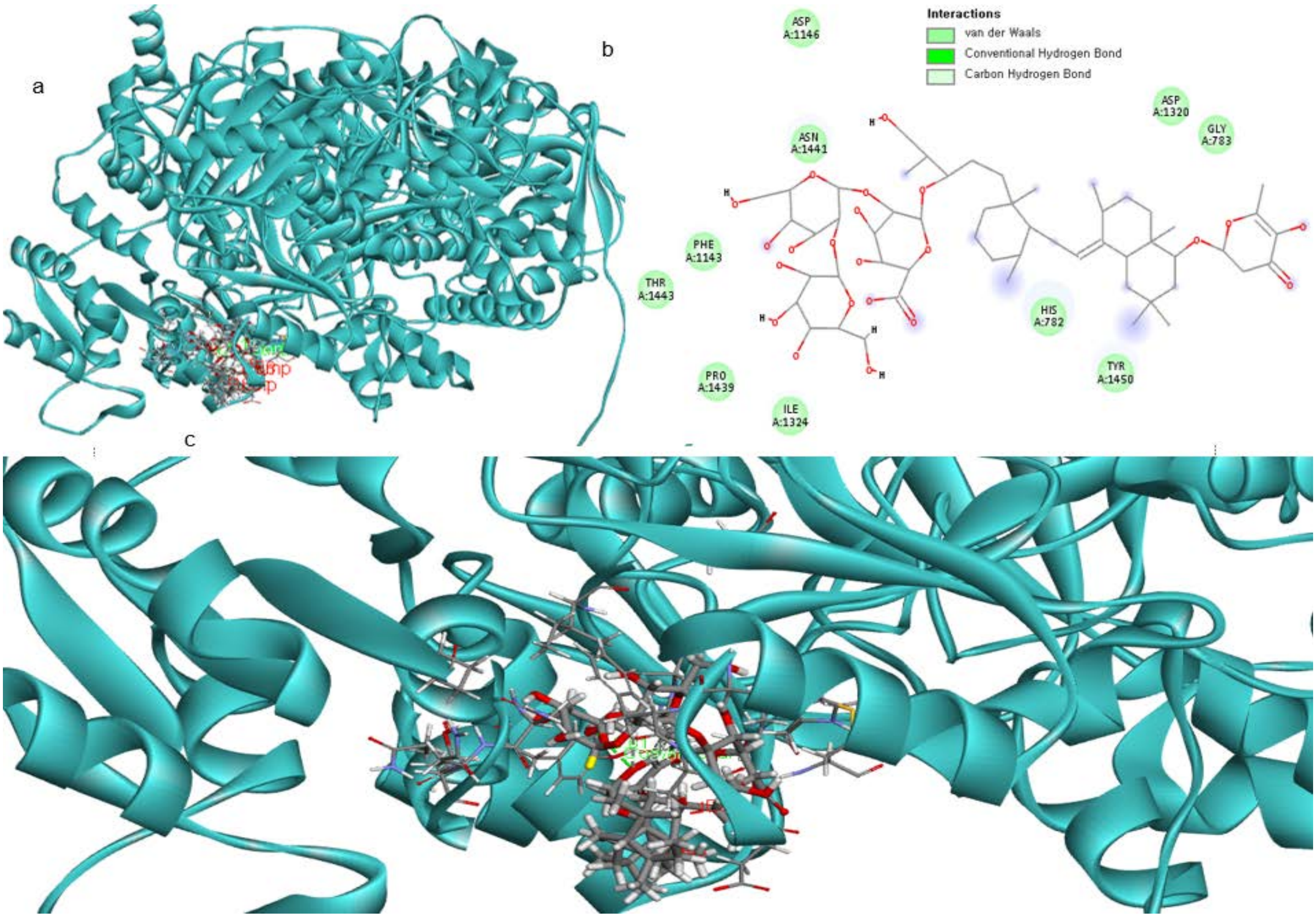
a
b
c
Interactions
van der Waals
Conventional Hydrogen Bond
Carbon Hydrogen Bond
ASP A:1146
ASN A:1441
PHE A:1143
THR A:1443
PRO A:1439
ILE A:1324
HIS A:782
TYR A:1450
ASP A:1320
GLY A:783

Figure S4. Molecular docking of CPS1 WT protein (Thr1406)(A) and MT (1406Asn)(B) with vitamin D3. Molecular docking with the bioactive compound and carbamoyl phosphate synthetase-1 (CPS1) wild-type protein (WT, Thr1406). (a) Diagrammatic representation of the compound (balls and stick model) binding with CPS1, (b) 2D depiction of CPS1 interacting with the compound and the nature of forces involved in stabilizing CPS1–vitamin D3-complex, and (c) Binding of the compound at the central cavity of CPS1.

A

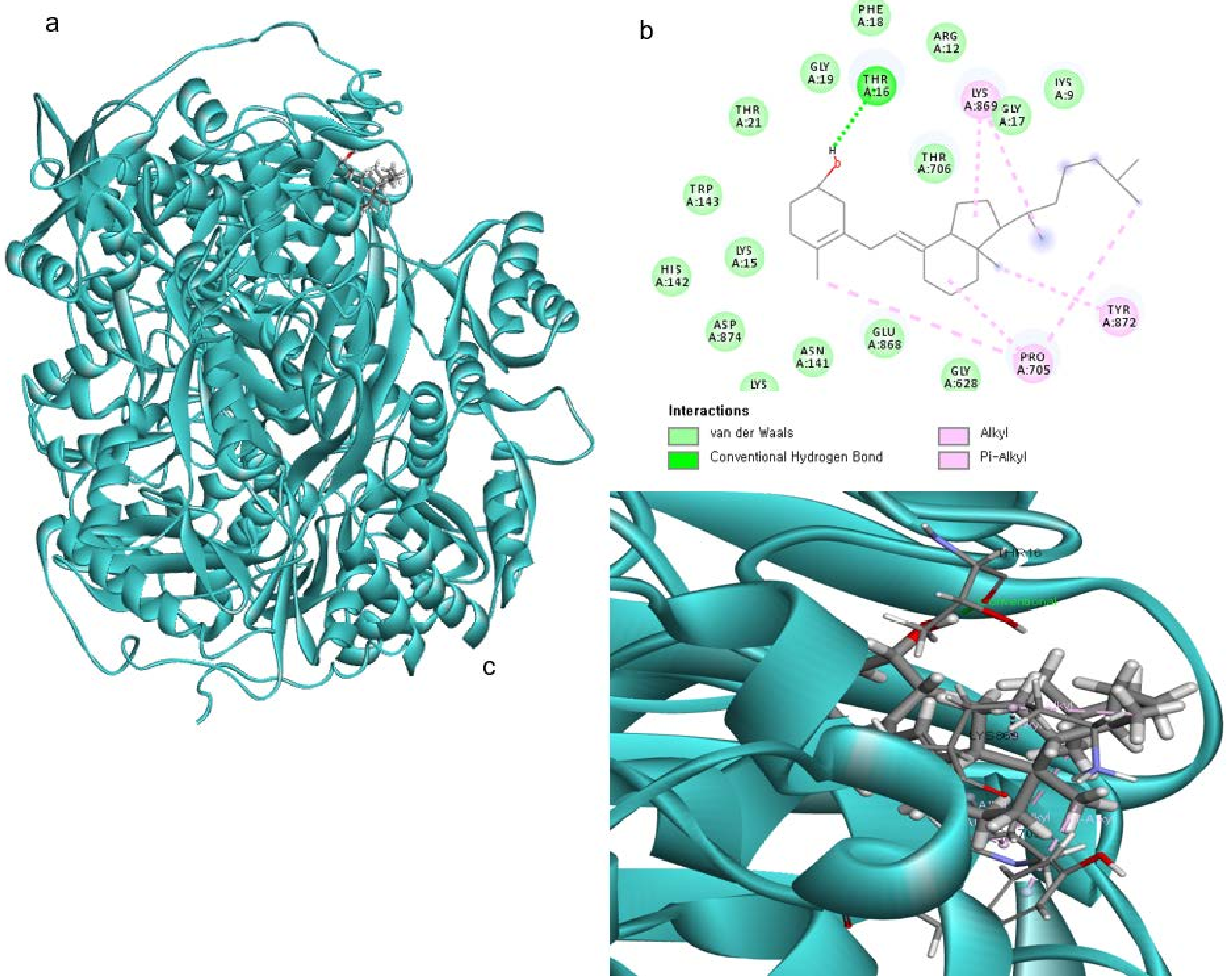

B

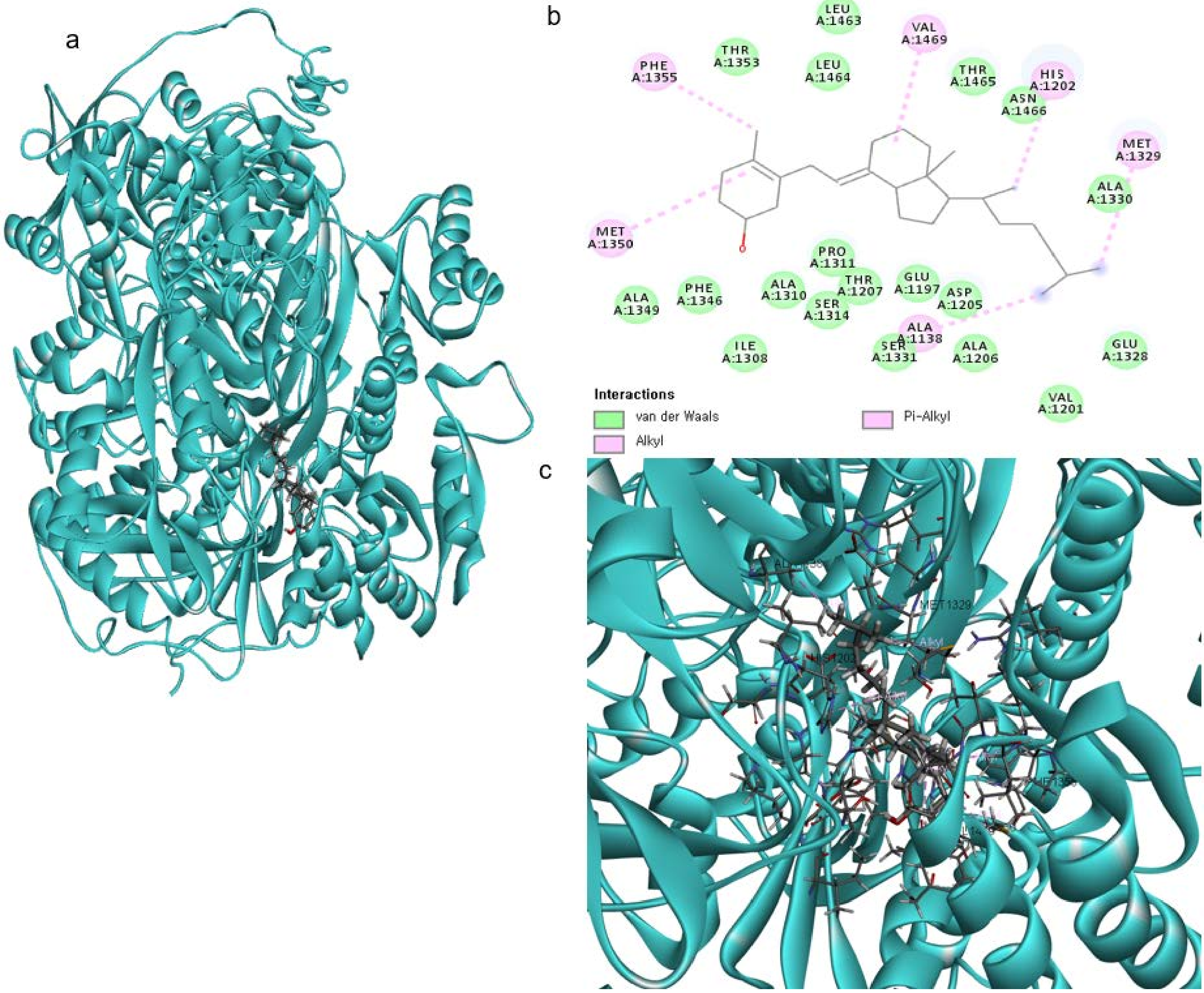
a
b
LEU A:1463
VAL A:1469
PHE A:1355
THR A:1353
LEU A:1464
THR A:1465
HIS A:1202
ASN A:1466
MET A:1329
ALA A:1330
MET A:1350
O
PRO A:1311
ALA A:1310
THR A:1207
GLU A:1197
ASP A:1205
ALA A:1349
PHE A:1346
SER A:1314
ALA A:1138
ILE A:1308
SER A:1331
ALA A:1206
GLU A:1328
VAL A:1201
Interactions
van der Waals
Alkyl
Pi-Alkyl
c
MET1329
